# Societal value of seagrass from historical to contemporary perspectives

**DOI:** 10.1007/s13280-025-02167-z

**Published:** 2025-03-24

**Authors:** Nicole R. Foster, Eugenia T. Apostolaki, Katelyn DiBenedetto, Carlos M. Duarte, David Gregory, Karina Inostroza, Dorte Krause-Jensen, Benjamin L. H. Jones, Eduard Serrano, Rym Zakhama-Sraieb, Oscar Serrano

**Affiliations:** 1 Centro de Estudios Avanzados de Blanes, Consejo Superior de Investigaciones Científicas, Blanes, Spain; 2 Institute of Oceanography, Hellenic Centre for Marine Research, PO Box 2214, 71003 Heraklion, Crete Greece; 3 Tennenbaum Marine Observatories Network and MarineGEO Program, Smithsonian Environmental Research Center, Edgewater, MD USA; 4 Marine Science Program, Biological and Environmental Science and Technology Division, King Abdullah University of Science and Technology, 23955-6900 Thuwal, Saudi Arabia; 5 Department of Conservation and Natural Science, The National Museum of Denmark, Copenhagen, Denmark; 6 BIOSFERA Research & Conservation, Girona, Spain; 7 Department of Ecoscience, Aarhus University, C.F. Møllers Allé, Building 1131, 8000 Aarhus C, Denmark; 8 Project Seagrass, Brackla Industrial Estate, Unit 1 Garth Drive, Bridgend, CF31 2AQ UK; 9 Seagrass Ecosystem Research Group, Department of Biosciences, Swansea University, Swansea, SA2 8PP UK; 10 Department of Earth and Environment, Institute of Environment, Florida International University, Miami, FL USA; 11 Faculty of Sciences of Tunis, Research Laboratory of Diversity, Management and Conservation of Biological Systems, University of Tunis El Manar, LR18ES06 Tunis, Tunisia; 12 High Institute of Biotechnology of Sidi Thabet, University of Manouba, BiotechPôle, BP-66, 2020 Sidi Thabet, Ariana Tunisia

**Keywords:** Cultural, Ecosystem services, History, Management, Social-ecological

## Abstract

Seagrasses have been entwined with human culture for millennia, constituting a natural resource that has supported humanity throughout this history. Understanding the societal value of seagrass fosters appreciation of these ecosystems, encouraging conservation and restoration actions to counteract historic and predicted losses. This study overviews the plethora of seagrass use in human history, ranging from spiritual and ceremonial roles, direct and indirect food resources, medicines and raw materials, dating back more than 180 000 years. While many past uses have been abandoned in modern societies, others have persisted or are being rediscovered, and new applications are emerging. As these uses of seagrasses depend on harvesting, we also underscore the need for sustainable practices to (re)generate positive interactions between seagrasses and society. Our review contributes to revalue seagrass societal ecosystem services, highlighting ancient and more recent human and seagrass relationships to incentivize conservation and restoration actions.

## Introduction

Seagrass meadows are coupled social-ecological systems that have sustained human well-being and livelihoods for thousands of years (Cullen-Unsworth et al. 2014; Nordlund et al. 2024). They provide a range of important ecosystem services including capturing and storing carbon, stabilising sediments, supporting marine and coastal food webs and serving as habitat for marine organisms (Nordlund et al. 2016; de los Santos et al. 2020). There are 72 species of seagrass worldwide, covering subpolar to tropical habitats (Short et al. 2011). Their distribution encompassing sheltered coastal environments, along with their ability to support extensive marine biodiversity, may help explain why human settlements often coincide with the distribution of seagrass habitat. The proximity to seagrass meadows has fostered cultural, spiritual and provisioning relationships with seagrass throughout human history and has led to seagrass featuring across human societies from past to present. Historically, seagrass meadows, and the seagrass species that occupy them, have been used for a variety of applications ranging from food resources, industrial materials and medicines to rituals and customs (Nordlund et al. 2016). Some of these applications remain undocumented or have been lost entirely, while others are still maintained by coastal indigenous and local communities today, or are being reinvented in the modern era. The concentration of human activities in the coastal zone–while fostering positive human seagrass relationships–is unfortunately a key driver for the increasing major global losses of seagrass ecosystems (de los Santos et al. 2019; Dunic et al. 2021).

**Fig. 1:**
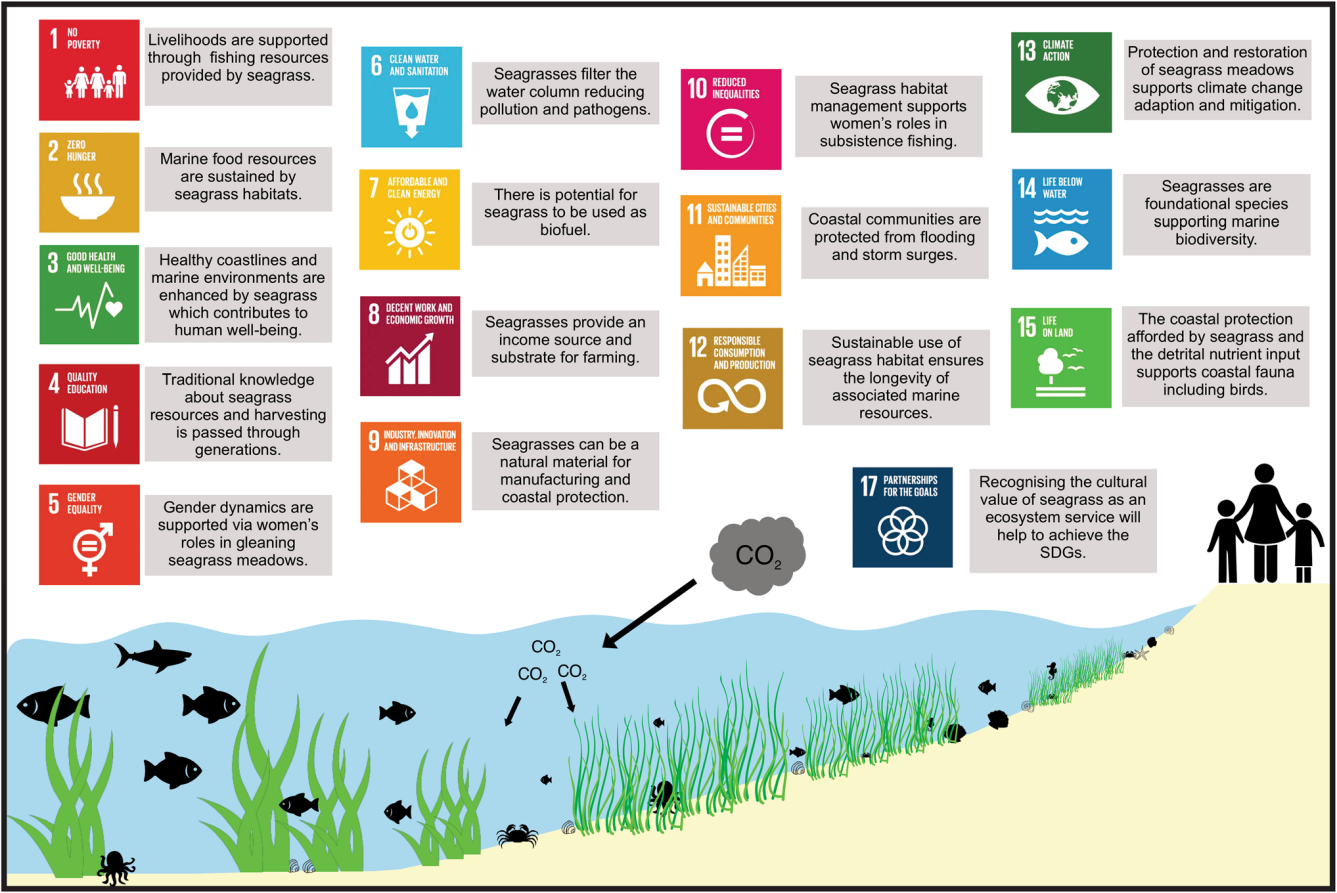
An infographic summarising seagrass contribution towards the Sustainable Development Goals. **(**Vector images obtained from vecteezy.)

As we envision moving away from current production models and consumption patterns towards ‘greener’ or circular economies, there is a drive to incorporate nature-based solutions to mitigate planetary crises. As such, the protection and restoration of seagrass meadows are continually being highlighted in relation to achieving multiple Sustainable Development Goals (SDG; Fig. 1) (Unsworth et al. 2022). In valuing the societal benefits of seagrass, we can work towards achieving these SDG’s. While harnessing seagrass for social or industrial applications can increase the visibility of seagrass in society, both raising awareness and rekindling humanity’s relationship with this resource, if not managed appropriately, it risks overexploitation and further loss of seagrass ecosystems.

**Fig. 2:**
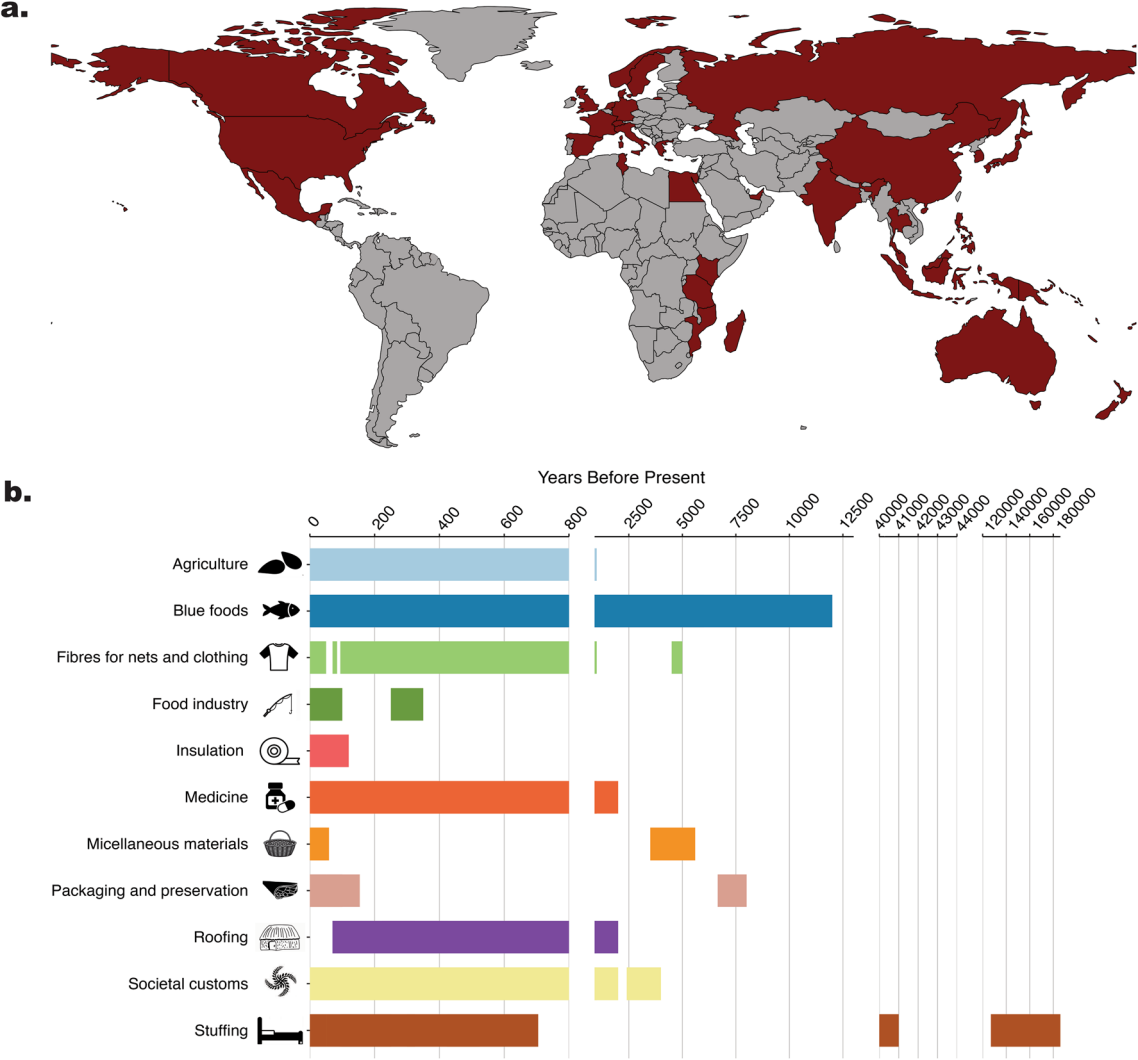
**a** The geographical distribution of documented cases of seagrass use in human societies that have been included in this review (coloured red by country).** b** An overview of direct uses of seagrass throughout history. Identified uses of seagrasses are listed on the x-axis. The y-axis depicts the range of Years (Before Present) that these uses were documented throughout history, where gaps represent a jump in the ages for plotting purposes, not missing data. (Vector images obtained from vecteezy.)

We have an opportunity to learn from our history, to understand past human and seagrass relationships, and the role seagrass has played in society. This is key to improving the way we value seagrass habitats. There is limited knowledge on the social aspects of seagrasses as an ecosystem service, which lessens the ability to assess their societal value (Ruiz-Frau et al. 2017). Here, we build on earlier reviews of direct uses of seagrasses (e.g. Pendergast et al. 2002; Milchakova et al. 2014; Ruiz-Frau et al. 2017), collating literature across multiple repositories (Google Scholar, Web of Science, eHRAF world cultures database, Google) and speaking to the seagrass research community, to present an updated and comprehensive synthesis of the available information for seagrass use in human society. We showcase how and where seagrass has featured in human history (Fig. 2), the diverse relationships between different societies and seagrasses, and how these relationships have changed throughout history. This can help assign greater emphasis on the societal and cultural value of seagrass, as an additional driver to help incentivise conservation and restoration efforts towards maintaining seagrasses for the future.

## Seagrass in food

### Seagrass as a direct food resource

Seagrasses are well established as sources of food for marine megafauna such as turtles and dugongs, but what is less well known is their historical role as a direct food source for humans. The seagrass *Zostera marina*, which is present along the temperate North Pacific, North Atlantic and Mediterranean bioregions (UNEP-WCMC and Short 2021), has been harvested by multiple coastal human communities throughout history. The Indigenous Kwakiutl peoples of Canada harvested *Z. marina* around 10 000 years ago by twisting a pole into seagrass meadows, bringing the leaves up and breaking them off near the rhizome. The roots and outer leaves were then removed, and the youngest inner leaf was wrapped around the rhizome and consumed by dipping in oil (Cullis-Suzuki et al. 2015). This practice was even discovered to maintain seagrass health by enhancing recruitment, genetic diversity and resilience (Cullis-Suzuki et al. 2015). The seeds of *Z. marina* were a source of food for the people of Isla Cedros, Baja California around 12 000 years ago (Fauvelle et al. 2012) and for the Seri people of Mexico around 2000 years ago. In Seri culture, *Z. marina* seeds were harvested and ground into flour that was then mixed with water to make a gruel or baked into bread and cakes (Felger and Moser 1973; Burckhalter 2021). While these specific practices have been preserved in Indigenous and local knowledge, post-colonialism losses of native languages and knowledge, and the wide distribution of *Z. marina*, suggest that similar consumption patterns likely existed across Europe and Asia, and extend much further back into human history.

**Fig. 3:**
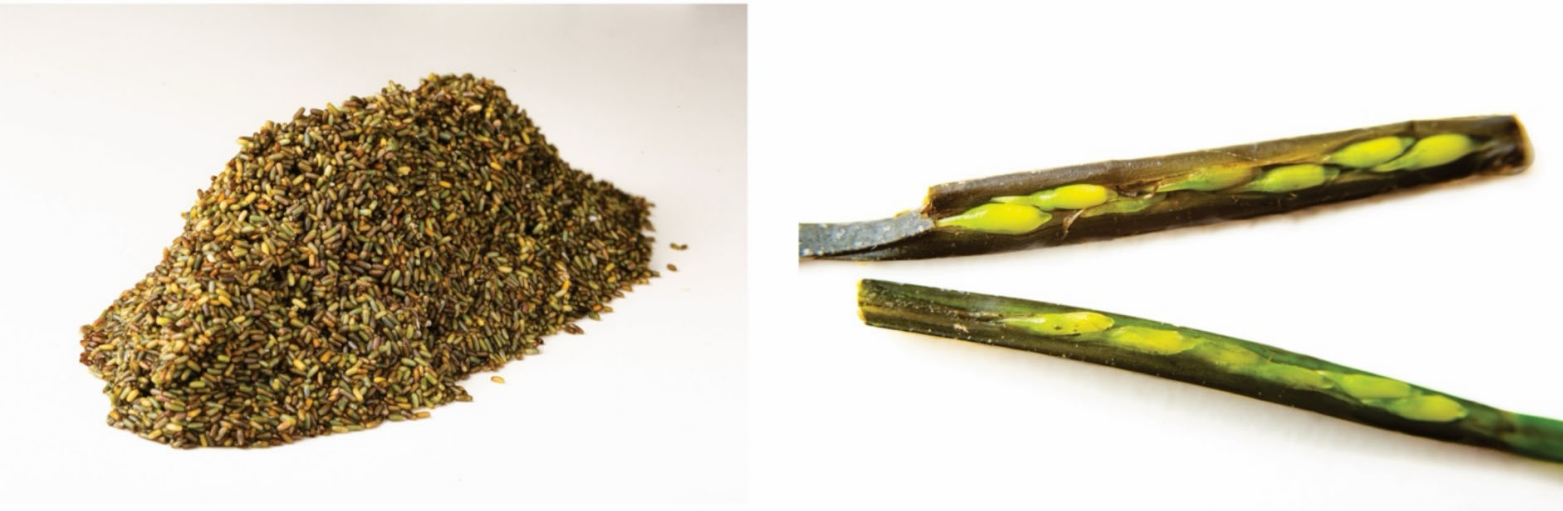
The seeds of *Zostera marina* are being used by the three Michelin star restaurant Aponiente in Spain. Photos provided with permission by the Aponiente restaurant

Learning from indigenous practices and in particular, those of the Seri people, the three Michelin star chef Ángel León from the restaurant Aponiente in Spain, has been experimenting with incorporating *Z. marina* flour into pasta, rice and bread (Fig. 3). This initiative has fuelled interest in production of *Z. marina* seeds, which do not require arable land, irrigation or fertiliser, leading to less methane emissions compared to rice production (Pérez-Lloréns and Brun 2023). Laboratory analyses have shown that *Z. marina* seeds contain nutritional properties (proteins, fats, carbohydrates) that are within similar ranges as other grains (rice, rye and durum wheat), supporting the use of seagrass as a possible alternative grain (Pérez-Lloréns and Brun 2023). Additional seagrass species have also been examined for the potential to use their seeds as food resources.

The large seeds of *Enhalus acoroides* are traditionally harvested by tropical coastal communities and were recently tested as a flour substitute, with studies showing similar nutritional properties to wheat, cassava and rice (Montaño et al. 1999). It was concluded, however, that large-scale production of *E.acoroides* seeds would be an issue, given the slow growth rate of this species and the amount of seagrass area required to produce enough seed (Montaño et al. 1999). Similar conclusions are likely for seagrasses in general. Harvesting and eating *E. acoroides* seeds is still common practice amongst fisherman in the Philippines (Montaño et al. 1999), in India–where *E. acoroides* is known as ‘Vattalai’ (Newmaster et al. 2011), in Thailand–where it is thought that consuming the seeds will bring good luck (Supanwanid and Lewmanomont 2003) and in the Maldives and Sri Lanka (C.M. Duarte, B.L.H. Jones personal observations), although there is no record of when this practice, likely very ancient, originated.

The fruits of *E. acoroides* are also harvested for food by the local people of Malaysia, Indonesia and the Solomon Islands (DSCP 2018; Nessa et al. 2020). It is common practice for the women of these communities to be the ones to collect the fruit, which serves as both food for people here and as a source of income if enough fruit is collected (Nessa et al. 2020). The rhizomes of *E. acoroides* have also been documented as a food resource, being consumed by people from the Lamu Archipelago in Kenya–here it is called ‘mtimbi’ (Ochieng and Erftemeijer 2003). Harvesting seagrass seeds, fruits and rhizomes for food is common practice amongst coastal communities throughout the world, emphasising the importance of seagrass as a food resource.

### Seagrass as an indirect food resource

The role of seagrasses as food for local communities extends beyond direct consumption as they provide habitat for marine organisms, facilitating an indirect food source. The Seri people of Mexico were known to collect seagrass leaves to consume the herring eggs that had been deposited (Felger and Moser 1973)–a similar practice was also reported in south-eastern Alaska by the Tlingit people (Emmons and De Laguna 1991). The Seri people also harvested waterfowl that grazed seagrass and hunted turtles that fed on seagrass, noting that their meat was sweeter than turtles that fed on algae (Felger and Moser 1973). In their role as habitat providers, seagrasses have historically supported fishing in many coastal communities throughout the world. Around 6000 years ago, the people of eastern Arabia relied on seagrass for hunting rabbitfish (Lidour et al. 2020) and in Fiji, people have depended on seagrass as fishing grounds for thousands of years (Cullen-Unsworth et al. 2014). There is even evidence of seagrass-associated molluscs (*Pecten* spp.) being collected by Neanderthals for food around 120 000 years ago (Colonese et al. 2011).

Today, local communities throughout the world focus fishing activities within seagrasses as their main source of food and income. This is documented within local fishing communities in Indonesia, Tanzania and the Solomon Islands (De La Torre-Castro and Rönnbäck 2004; Cullen-Unsworth et al. 2014; McKenzie et al. 2021), and across the Indo-Pacific more broadly, where seagrasses were found to be more reliable than coral reefs for collecting fish and invertebrates (Jones et al. 2022). Seagrass fishing activities in these regions include invertebrate gleaning, which depends heavily on seagrass beds and is predominantly undertaken by women (Ratnawati et al. 2019; Chitará-Nhandimo et al. 2022; Stiepani et al. 2023). A survey of a local gleaning community in Zanzibar reported declining invertebrate abundances in recent years, owing to the reduction in seagrass habitat, increasing algal coverage and changing species assemblages in seagrass beds–all linked to overfishing in the area (Stiepani et al. 2023). This is threatening both the food security and livelihoods of communities who rely on gleaning.

### Seagrass in the food industry

Seagrasses can further support livelihoods and income by providing food and habitat for farming. One example is the sea cucumber industries in the Solomon Islands (McKenzie et al. 2021) and Australia (Kendrick et al. 2019), which are supported by seagrass habitat. This was Australia’s first recorded export and is still alive today, with harvesting being undertaken by the Indigenous owned company Tidal Moon based in Shark Bay (Western Australia). Noteworthy, the marine heatwave that devastated 1000 km^2^ of seagrass in this marine World Heritage Site had a major impact on sea cucumber fisheries (Kendrick et al. 2019). Seagrass has also been recorded as food for livestock such as in the 1700s in Norway, when *Zostera* spp*.* was used to feed cows; the story goes that cows were observed wading out to eat the seagrass and so it was collected and stored to provide food for them during winter (Alm 2003). Another example of this is in India where local communities use *E. acoroides* seeds in feed for goats, pigs and cattle (Newmaster et al. 2011). Even today, seagrass is incorporated into livestock supplements by companies such as Beachport Liquid Minerals in Australia. These applications reiterate the important role seagrasses play as a food resource both today and throughout history.

## Seagrass in material applications

### Seagrass as roofing

**Fig. 4:**
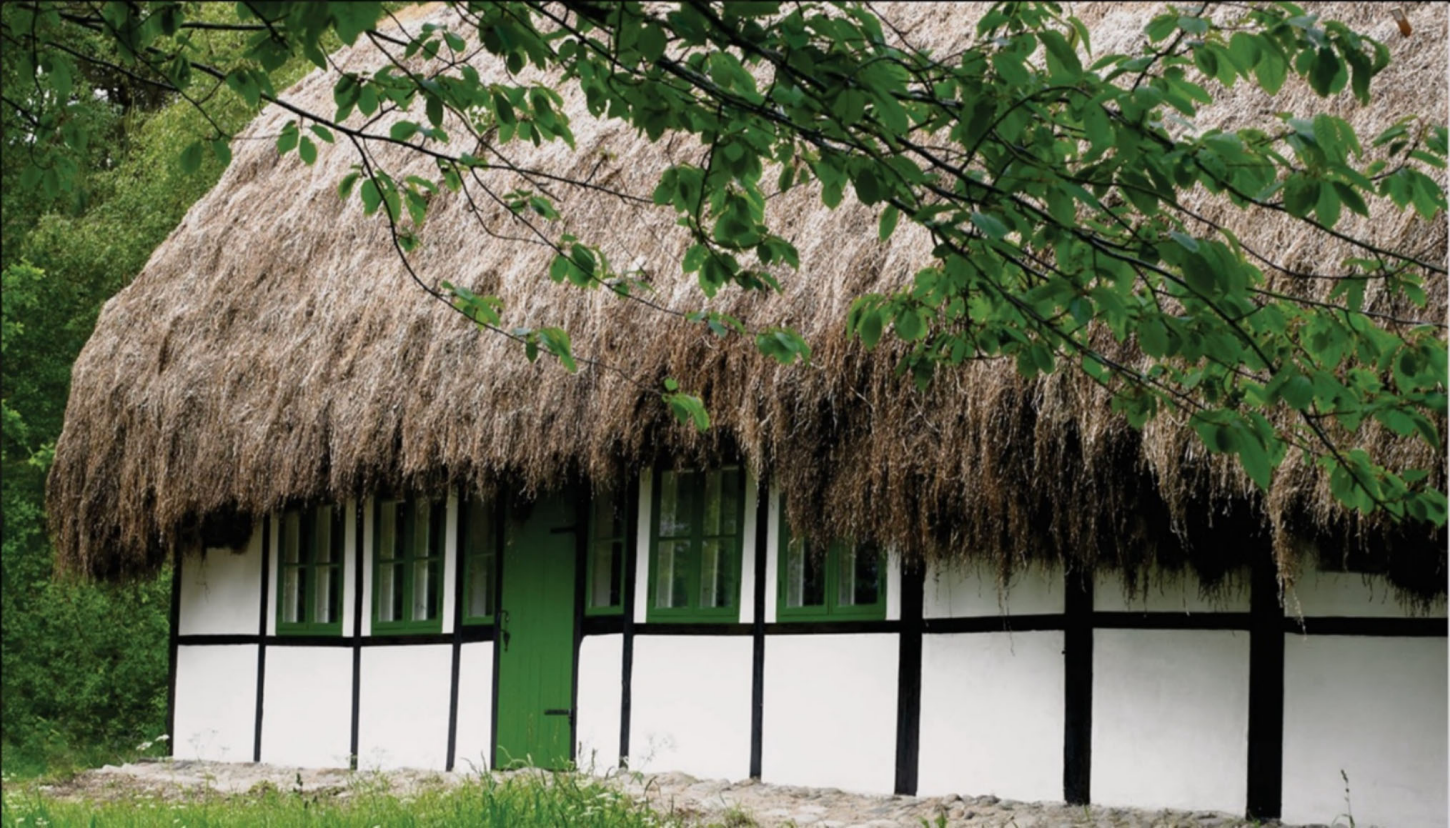
A traditional thatched roof made of *Zostera marina* in Læsø Denmark dated to 1865. Photo provided with permission by Helene Hoeyer Mikkelsen / Realdania By & Byg

Seagrasses have been utilised in numerous material applications throughout human history, notably the leaves of seagrass have been used for roofing purposes across multiple human cultures. The Seri people in Mexico utilised seagrass as roof thatching around 2000 years ago (Felger and Moser 1973) and this was also documented in Sweden (Linné 1745; Alm 2003) and Great Britain (Wyllie-Echeverria et al. 2000) in the 1700s, as well as in China (1000 years ago), where the practice is still maintained in some villages today (Liu et al. 2023). A special practice of seagrass roofing dating back to the 1600 s on the Danish island Læsø (Lockley 1952) (Fig. 4) has recently been rediscovered^1^. Inspired by a wish to restore the old roofs and maintain this cultural heritage, this practice has placed Læsø on the tentative list of UNESCO World Heritage. Roofs made of seagrass are said to be durable and insulating (Liu et al. 2023), lasting up to 200–300 years (Lockley 1952). The antimicrobial, antiviral and antiparasitic properties of seagrass work to prevent decay, justifying the enduring nature of seagrass roofs and explaining their widespread use across different human cultures (Orhan et al. 2006; Hengrui et al. 2014; Acharjee et al. 2021).

### Miscellaneous materials using seagrass

The use of seagrass as a material product is recorded in various other applications in human history. The seagrass *Posidonia* spp. was documented in mud bricks from Crete, Greece around 1500 years ago (Devolder and Lorenzon 2019; Lorenzon 2021) and recent tests have shown the unique strength and structure of lignin in *Posidonia* spp*.*, opening possibilities to genetically modify lignin in trees to improve wood materials in modern buildings (Rencoret et al. 2020). Seagrass can even be made into paper given its high cellulose content and tests have shown *E. acoroides* would work best for this purpose (Syed et al. 2016). It has also been documented as packing material for glassware across the Mediterranean in the 1970s, where it was known as ‘Venetian straw’ (Alm 2003; Vasarri et al. 2021). More modern material uses have been discovered for seagrass such as in wall coverings and veneers for furniture and cupboards, which are sold by the European company Metis Seagrass. There are also accessories available, such as phone cases and sunglasses made from dried *P. oceanica* leaves by the Greek company Phee.

### Packaging and preservation using seagrass

The material applications of seagrass even extend to packaging material for food preservation and in basketry. Traditionally, seagrass was used to pack crabs in the Chesapeake Bay (Milchakova et al. 2014) and nowadays it is used in fishing markets in India to keep fish cool in summer (Newmaster et al. 2011). The antimicrobial properties of seagrass explain their use in food preservation, with tests on *P. oceanica* demonstrating seagrass can be a sustainable method to reduce spoilage (Vasarri et al. 2021) and preserve red meat (Benito-González et al. 2021). These properties are likely why seagrass was historically used to make baskets. In the 1870s, the North American Makah people wove baskets from seagrass leaves, and these were white because the leaves of *Zostera* spp. bleach in the sun when they dry (Swan 1870). Similar baskets made from *Zostera* spp. were discovered off the coast of California and are believed to be around 8000 years old (Connolly et al. 1995). Alongside the baskets were ropes, sandals, fishing lines and nets, all made from *Posidonia* spp*.* fibres (Vellanoweth et al. 2003).

### Seagrass fibres for nets and clothing

Using seagrass fibres to produce fishing equipment has been documented across multiple societies. In Japan, *Phyllospadix* spp. was historically made into rope for fishing (Aioi and Nakaoka 2003) and in the present day, the seagrass, *E. acoroides* is made into lures and nets in Micronesia and the Solomon Islands (McKenzie et al. 2021). The versatility of seagrass also extends to clothing where it has been documented in the mourning caps of the Maori people of New Zealand (Inglis 2003), in footwear in ancient Egypt (Vasarri et al. 2021) and in Japan to make wet weather gear until the 1930s when rubber was introduced (Milchakova et al. 2014). In fact, seagrass is said to have been used to cover army uniforms of French soldiers in WWI as camouflage; ‘Eelgrass leaves were used to garnish grass-like camouflage mesh; their rot-proof and non-flammable nature making them preferable to any other product for this purpose’ (Lami 1941). Evidently, seagrasses are a multi-purpose, natural material, serving a range of applications throughout human history.

### Seagrass as stuffing material

Seagrasses have also been recorded in stuffing for mattresses and upholstery where these applications are as old as human society. Almost 200 000 years ago in France, seagrass was discovered in the bedding of early humans, and this was observed again around 40 000 years ago in Italy (Colonese et al. 2011). Seagrasses are seemingly an effective bedding material as this have been recorded across multiple human societies such as in South Africa in the 1700s (Inskeep 1978), in the Netherlands, Great Britain and the United States in the 1800s (Wyllie-Echeverria et al. 2000), in Russia in the 1950s (Milchakova et al. 2014), and in a psychiatric ward in Switzerland in the 1900s (Luchsinger 2020). Font Quer (1976) reports, in his book on medicinal uses of plants, that Pope Julius III, in the 1500 s, requested a *Posidonia*-stuffed mattress as it was shown to provide protection against bed bugs and other pests. Seagrass is even still being used in mattresses today, with companies such as Coco-mat (Spain) incorporating seagrass leaves into the stuffing of their mattresses.

The use of seagrass leaves as an insulating material featured in large historical industrial practices. In the 1800s, seagrass was harvested commercially in the United States, Canada, Norway, Sweden, Netherlands, France and Great Britain (Wyllie-Echeverria et al. 2000). Of note, was the seagrass harvesting industry in the Netherlands, which incorporated seagrass leaves into mattresses and pillows. Harvesting of seagrass in this region was undertaken freely until 1844 but by 1898, this became heavily regulated and there were severe punishments for illegally harvesting seagrass. This industry thrived up until 1927, when the construction of the Afsluitdijk–a 32 km long dike installed to prevent flooding–led to major seagrass die back (van Eerbeek 2010) and nearly all of the 15 000 hectares of seagrass in this area disappeared. A morphotype of *Z. marina* which was instrumental to the industry here became locally extinct, bringing about the downfall of this industry (van Katwijk et al. 2024). Ironically, seagrass was also used as a material for historical sea dike construction in the Netherlands, with one record dating back to 1319 (van der Meer 2009).

### Seagrass in insulation

**Fig. 5:**
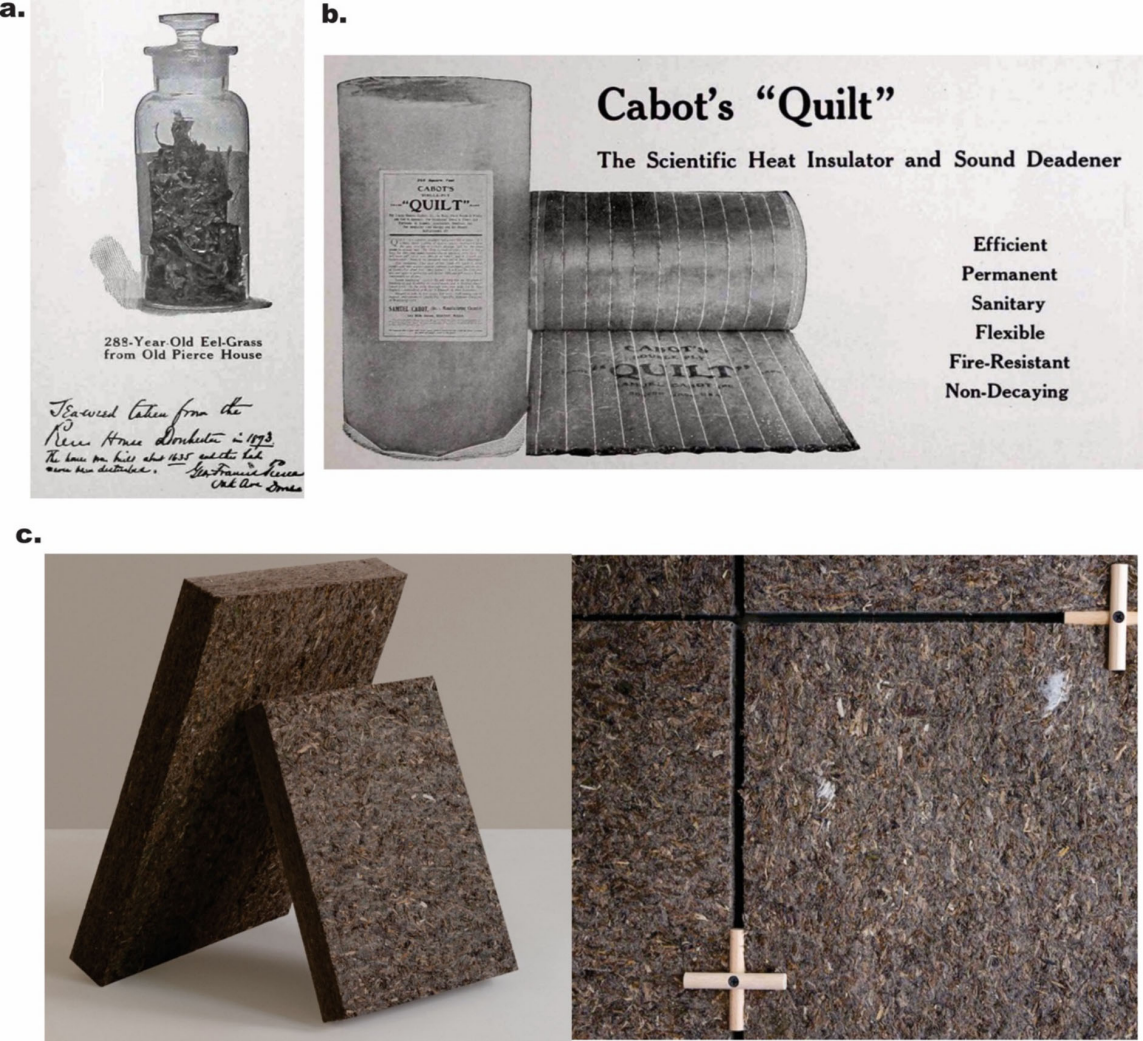
Seagrass as insulating material.** a** 288-year-old seagrass *Zostera marina* used as evidence for the longevity of Cabots Quilts.** b** Cabots Quilts advertisement from a 1923 brochure. (Cabot 1923).** c** Acoustic mats made by the company Søuld using *Zostera marina*. Photos reproduced with permission from Søuld

Another large seagrass industry was the harvesting of seagrass for insulation in Nova Scotia, Canada. Here, harvesting continued into the 1900s and was run by the company Cabots Quilts (Fig. 5a, b). Dried leaves of *Z. marina* were used to make insulation quilts (Wyllie-Echeverria and Cox 1999), which were even used by the first American and British expeditioners to visit the Antarctic (Kirkman and Kendrick 1997). These quilts were also described as sound insulating as a note accompanying a quilt found in Great Britain stated it was ‘Used for deadening sound in the new London Library and elsewhere’ (Pendergast 2002). Unfortunately, seagrass wasting disease decimated the seagrass population in Nova Scotia, leading to the collapse of Cabots Quilts in the 1930s. By the 1950s the meadows and the industry had recovered, but the industry closed again in the 1960s when fibreglass became the preferred material for insulation (Wyllie-Echeverria and Cox 1999).

A similar industrial pursuit was undertaken in Australia in the early 1900s when *Posidonia australis* was harvested off the coast of Southern Australia. This was initiated when a link was made between *P. australis* fibre balls washing up on shore and the marine fibre deposits of *P. australis* (Pendergast 2002). Harvesting then began to dredge these marine fibre deposits from the sea floor (Pendergast 2002). These fibres were turned into an insulation product and some of the old government buildings in Australia still contain seagrass insulation from this period (Kirkman and Kendrick 1997, Seddon and Murray‐Jones 2002, Coles et al. 2003). This industry was eventually abandoned as it proved uneconomical (Womersley 1984), largely due to the devaluing of the fibre as it was said to be weak and brittle, and the limited use of seagrass for insulation in Australia (Pendergast 2002). Another seagrass harvesting industry began in Australia in 1970, when *Zostera muelleri* was harvested from the beaches in Western Port Bay, Victoria for use in insulation. This practice was abandoned shortly after due to declines in the species, which were eventually attributed to pollution in the area (Kirkman and Kendrick 1997).

Today, there is a resurgence of seagrass use in insulating materials such as those being sold by the company NeptuTherm who use *P. oceanica* fibre balls, and the company Søuld, who make acoustic mats from *Z. marina* (Fig. 5c). The seagrass, *P. oceanica* is also being trialled in a housing project in Spain where wrack is collected in areas where it over-accumulates and incorporated into insulation instead of being thrown into landfill. These records of seagrass use as insulation both in the present day and historically, demonstrate the potential of seagrass as a natural material for this purpose. If seagrass is to continue to be used in this way, there is a need to consider how this can be pursued sustainably, given the limited success of seagrass industries historically.

### Seagrass in agriculture

The nature of seagrass and inherent properties are why seagrasses have persisted in material applications throughout history. Seagrasses can concentrate minerals and elements (Milchakova et al. 2014) and readily uptake nitrogen and phosphorus (Touchette and Burkholder 2000), which not only means that they are good at reducing pollution (Udy et al. 1999; Fernandes et al. 2009) but also that they are useful for a variety of products, including in agriculture. Seagrass (*Z. marina*) was used as fertiliser in Norway in the 1700s (Alm 2003) and was previously harvested in Australia and Japan for compost (Aioi and Nakaoka 2003; Coles et al. 2003). Seagrass has also been applied as fertiliser for growing plants by the local people of Zanzibar, Fiji, the Solomon Islands and Madagascar (De La Torre-Castro and Rönnbäck 2004; DSCP 2018; McKenzie et al. 2021). In Tunisia, *P. oceanica* wrack is still being used as fertiliser in agriculture (Procaccini et al. 2003). A study on the use of *P. oceanica* found that it had potential as a sustainable fertiliser for use on crops such as tomato and lettuce (Grassi et al. 2015). Indeed, projects have proposed turning seagrass wrack into fertiliser, focusing on areas where the wrack accumulates in excess due to altered hydrodynamics along the coastline. The company Compost Hellas is doing just that, creating fertiliser products from *P. oceanica* wrack in Greece. There have also been proposals to convert seagrass wrack into biofuel (Masri et al. 2018) or into biochar, which can then be used to improve soil health (Macreadie et al. 2017).

Certainly, seagrass is used for many different products, and there is even a perfume that harnesses the aquatic marine scent of *Posidonia* spp*.,* made by the Italian brand Bulgari. This versatility is perhaps why seagrass has featured in diverse applications throughout human history. By harnessing the ecological, physical and chemical properties of seagrass, human societies have benefited from healthy seagrass meadows. In this way, the prosperity of many human communities is linked to the state of seagrass habitats.

## Seagrass in medicine

Healthy seagrass meadows can further ensure the well-being of coastal communities through medicinal applications. Historically, seagrasses were used in ancient Egypt to cure sore throat and skin problems (Vasarri et al. 2021) and by the Seri people of Mexico to treat diarrhoea (Felger and Moser 1973). Today, the leaves of *E. acoroide*s are used in the Solomon Islands to remove pain from fish stings (McKenzie et al. 2021) and as a mosquito repellent in Indonesia, where they are pounded with water and then applied as a dressing to the body (Noor et al. 2022). The roots of this species are also used by the Chwaka people of Zanzibar to treat muscle pain, wounds, and stomach problems (De La Torre-Castro and Rönnbäck 2004). In India, local people use seagrass to treat dandruff; the fresh leaves of *Halophila ovata* are ground into a paste and applied onto the scalp for a week (Newmaster et al. 2011). Similarly, a handful of ‘Elai pasi’ (*H. ovata*) leaves, mixed with sesame oil, are consumed for three days as a treatment for iron deficiency (Newmaster et al. 2011).

Using seagrass to treat more serious health problems has been documented elsewhere. Seagrasses *E. acoroides, Thalassia hemprichii, Cymodocea* spp., *Halophila* spp*.*, and *Thalassodendron ciliatum* are all used by the Chwaka people of Zanzibar in the form of Mafusho (‘smoke produced from burning seagrass’) to reduce fever and treat malaria (De La Torre-Castro and Rönnbäck 2004). Various skin diseases, burns and boils are treated in India using the leaves of ‘Murungai pasi’ (*H. ovalis*) (Newmaster et al. 2011) and in Zanzibar using a mixture of *Thalassia* spp. and *Cymodocea* spp. (De La Torre-Castro and Rönnbäck 2004). In India, for medical conditions such as heart disease and low blood pressure, people peel off the surface layer of the rhizome and eat the fresh rhizome of ‘Olai pasi’ (*E. acoroides*) with a cup of seawater (Newmaster et al. 2011). In Tanzania, seagrasses have traditionally been used as a remedy against typhoid fever. The seagrasses *Halodule uninervis* and *Cymodocea serrulata* were shown to exhibit antimicrobial properties against the bacterium *Salmonella typhi,* which causes typhoid fever (Hamisi et al. 2023).

Further studies have examined the chemical properties of seagrasses for potential medical applications. Findings revealed that *C. serrulata*, *H. ovalis* and *Zostera capensis* all possess antimicrobial properties (Kumar et al. 2008) which were shown to be successful in the treatment of urinary tract infections (Ragupathi Raja Kannan et al. 2012). In one study, researchers found *Cymodocea nodosa* contained both antimicrobial properties and cytotoxic activity against lung cancer cells (Kontiza et al. 2005). Leaves of several seagrass species (*E. acoroides, T. hemprichii, Halodule pinifolia, Syringodium isoetifolium, C. serrulata* and *Cymodocea rotundata*) were ground into a powder and tested for their nutritional properties with overall findings suggesting that they were high in protein, lipids and fibre, and could be used to treat diabetes (Rengasamy et al. 2013). There is even a product on the market today called ‘Zosterin ultra’ which is a powder containing Zosteran pectin, produced from *Z. marina*. This supplement is promoted as a treatment for peptic ulcer disease, to heal ulcers, to restore pancreatic function and improve metabolism.

The medicinal applications of seagrass reveal inherent properties that can be important for human health. Harnessing seagrass for this purpose both traditionally and today, highlights the prevalence of seagrass in human culture and customs that have persisted for thousands of years.

## Concerns around modern-day seagrass use

The many uses of seagrass throughout history emphasises their importance to human societies and captures the versatility of seagrass as a raw material. Given this, there is potential that seagrass will be used in our modern-day economy, especially with the current move to eco-friendly or ‘green’ alternatives, and not discounting the ‘Blue Acceleration’ that is, the trajectory of human expansion into the ocean (Jouffray et al. 2020). Yet, this may come at a cost for seagrass habitats globally. Humans have a propensity for overconsumption and destruction and as we have observed, we undertake industrial pursuits at the cost of the environment. This is evidenced by the major global declines of seagrasses over the past centuries caused largely by human pressures in the coastal zone (Waycott et al. 2009; de los Santos et al. 2019; Dunic et al. 2021). Our current economic system, based on growth, also poses issues for seagrass harvesting for industry on a large scale.

**Fig. 6:**
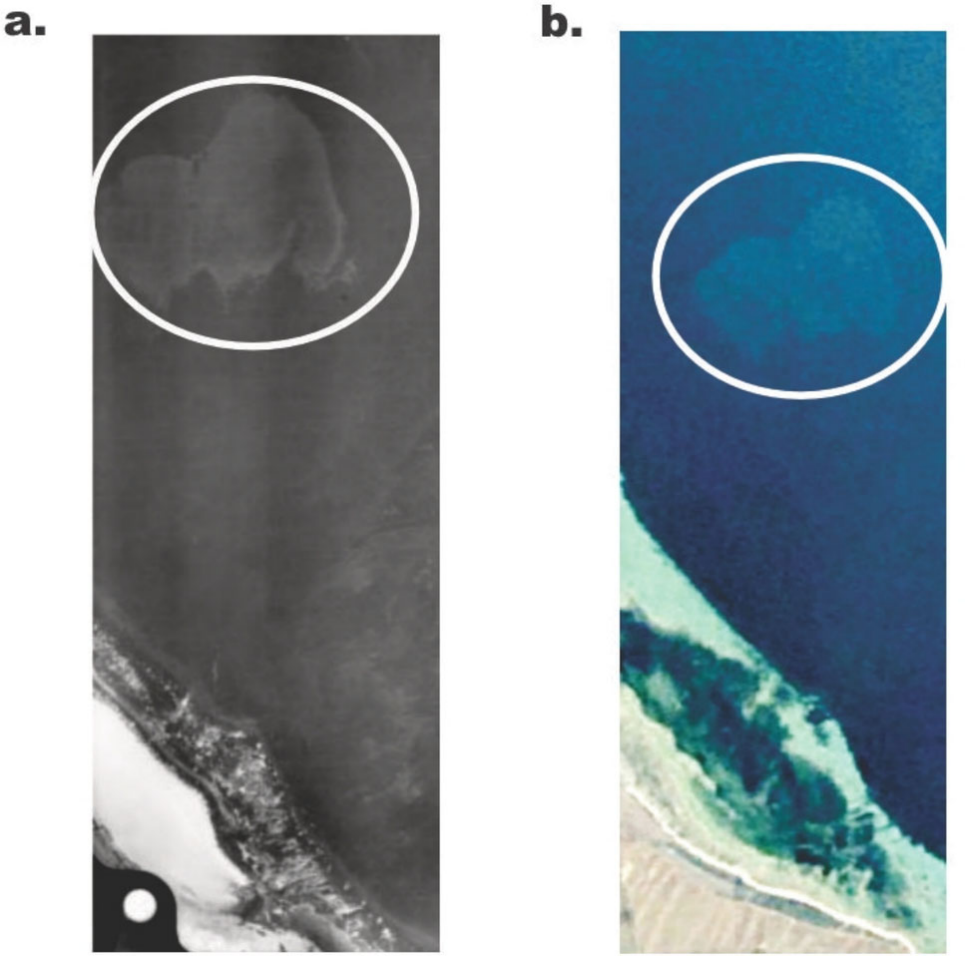
Evidence that large-scale removal of seagrass habitat for industry may never recover. (**a**) Image extracted from Kirkman (1997) which identified the seagrass harvesting at Port Broughton, South Australia. (**b**) Image obtained from Google Earth Pro in 2024 showing the seagrass scar was still present 62 years later in 2019. White circle indicates the scar in both images. (Image © 2019 Google Earth, Landsat/ Copernicus)

If seagrass is going to have a place in industry, whether this be food, building material, housing products or medicine, then it will need to be strictly regulated. The slow recovery of seagrass meadows implies that industrial pursuits need to be gentle and coupled with restoration of existing meadows. There is solid evidence that the mass harvesting of seagrass can cause irreparable damage. The harvesting of *P. australis* fibres in Southern Australia led to the removal of a large portion of seagrass and this is yet to recover since harvesting finished in 1914 (Fig. 6). There are existing projects that are opting to use seagrass wrack as a more sustainable alternative to harvesting seagrass and are doing so in areas where the wrack would otherwise be thrown into landfill. While this can be a better option to widescale harvesting, it still requires careful consideration of the ecological role of beach wrack and whether removal is necessary (Manfra et al. 2024). Without regulation of seagrass harvesting, we could lose valuable seagrass habitat which will impact marine organisms and ecosystems supported by seagrass and reduce contributions towards the SDGs. Yet, rediscovering the potential of seagrass for modern industry is a way to build awareness of this important habitat. If undertaken sustainably, this can encourage people to reconnect with seagrass to enhance human well-being.

## Seagrass in societal customs, practices, cultural heritage and human well-being

**Fig. 7:**
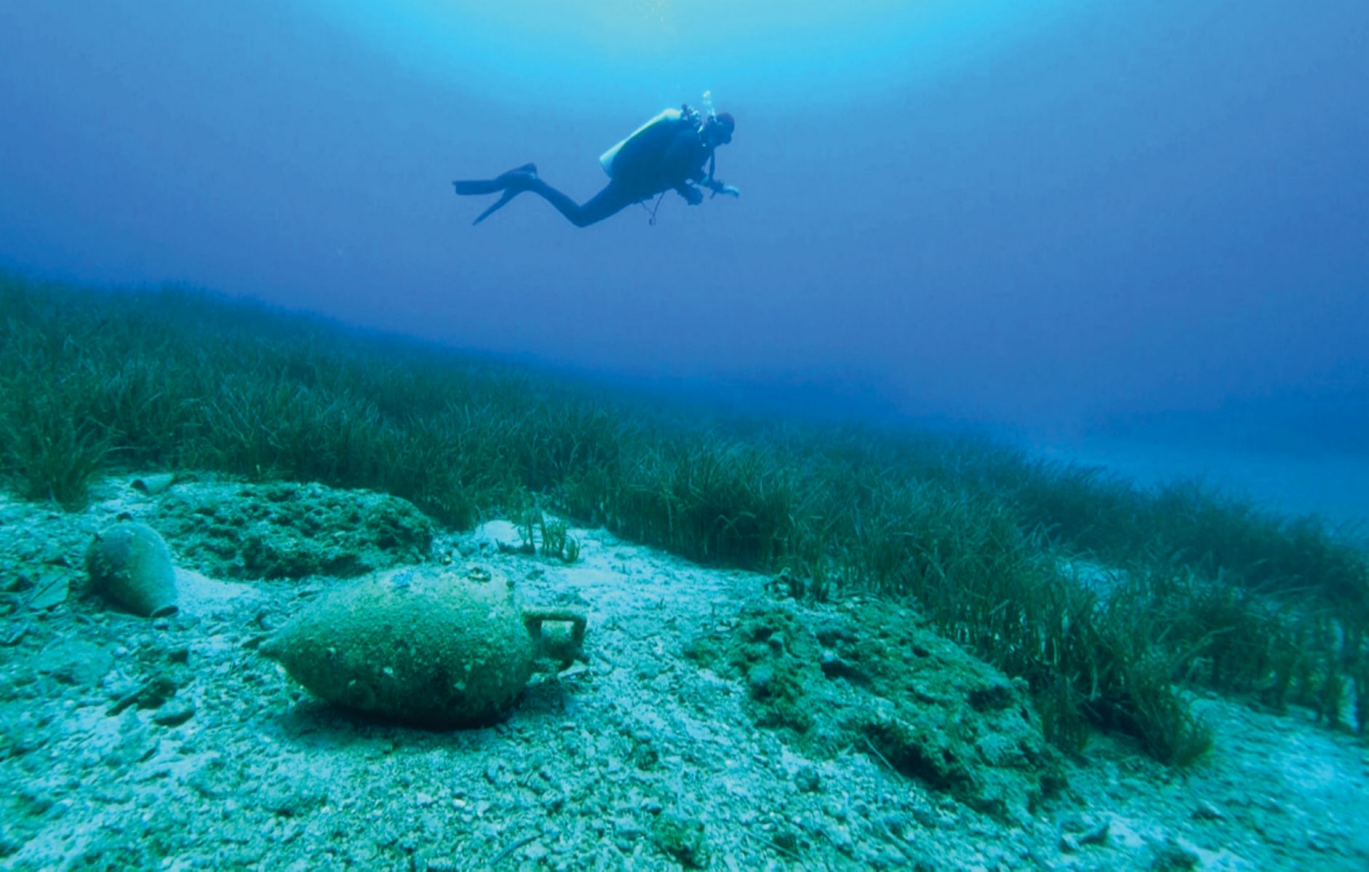
Photograph of historical human artefacts dating back more than 2000 years ago preserved in *Posidonia oceanica* meadows in Greece. Photo credit: Julius Glampedakis

Interconnectedness between people and seagrass can be further enriched by understanding instances where seagrass has had a role in human cultural beliefs and practices. In the Seri culture, seagrass was known as ‘wheat of the sea’ and April was the ‘moon of the seagrass seed’ indicating the best time for harvesting *Z. marina* seeds (Felger and Moser 1973; Burckhalter 2021). There is also mention of seagrass in the culture of native American, Pomo people as during weddings, when the bride’s family paid the groom's family back for the wedding feast, they paid with ‘beads, baskets, pinole, buckeye, acorn mush, **seagrass**, and acorn bread’. This same text describes women’s menstruation where women were not allowed to eat ‘meat, bird, or fish’ but were allowed to eat ‘mussels, kelp, **seagrass**, acorn bread, and pinole’ (Loeb 1926). Seagrass (*Z. marina*) was found in the foundation of many burial mounds of the Bosporus Kingdom in the Kerch Peninsula, Crimea (Milchakova et al. 2014) and in graves in Denmark during the Early and Late Bronze Age (Tornberg et al. 2022). Here, seagrass was used as packing around the coffins and urns, and it was thought the dead were wrapped in this seagrass to represent their close connection with the sea (Tornberg et al. 2022). Seagrasses themselves can even directly preserve human history and culture, acting as time capsules (Fig. 7). Their dense root structures trap and hold sediments, forming sedimentary archives of historical environmental data and in the process, they can preserve archaeological artefacts (Krause-Jensen et al. 2019).

Beyond being archives of the human past, seagrasses also feature in rituals and customs across human cultures. Evidence from the Red Sea suggests that opercula from seagrass-associated gastropods were burned to create incense for ceremonial purposes (Carannante et al. 2022). Moreover, molluscs of the family Muricidae, many of which are found in and associated with seagrasses, were a source of purple dye known as Royal purple and Tyrian purple, and were commonly used for garments for the priesthood, being specifically mentioned in the Holy Bible. Recent evidence suggests that opercula from these molluscs is also ‘onycha’, one of the four major ingredients of sacred incense (Nongmaithem et al. 2017). Local people in Zanzibar use seagrass in rituals and cures against ghosts and devils. Amulets and small packages containing seagrass are placed in boats (‘to always have the wind with you’) and in necklaces for babies to ward off nightmares (De La Torre-Castro and Rönnbäck 2004). Fishermen here also use *E. acoroides* for navigation, noting that it points out the direction and intensity of currents, and that the amount of seagrass in the water and washed up on beaches indicates seasonal changes (De La Torre-Castro and Rönnbäck 2004). In the Solomon Islands, fishermen use *E. acoroides* to call seagrass spirits to bring them good fortune with their catch. Tying the fibres of this seagrass into knots is thought to be a way to attract a mate or ensure a newborn child will be gifted (McKenzie et al. 2021).

**Fig. 8:**
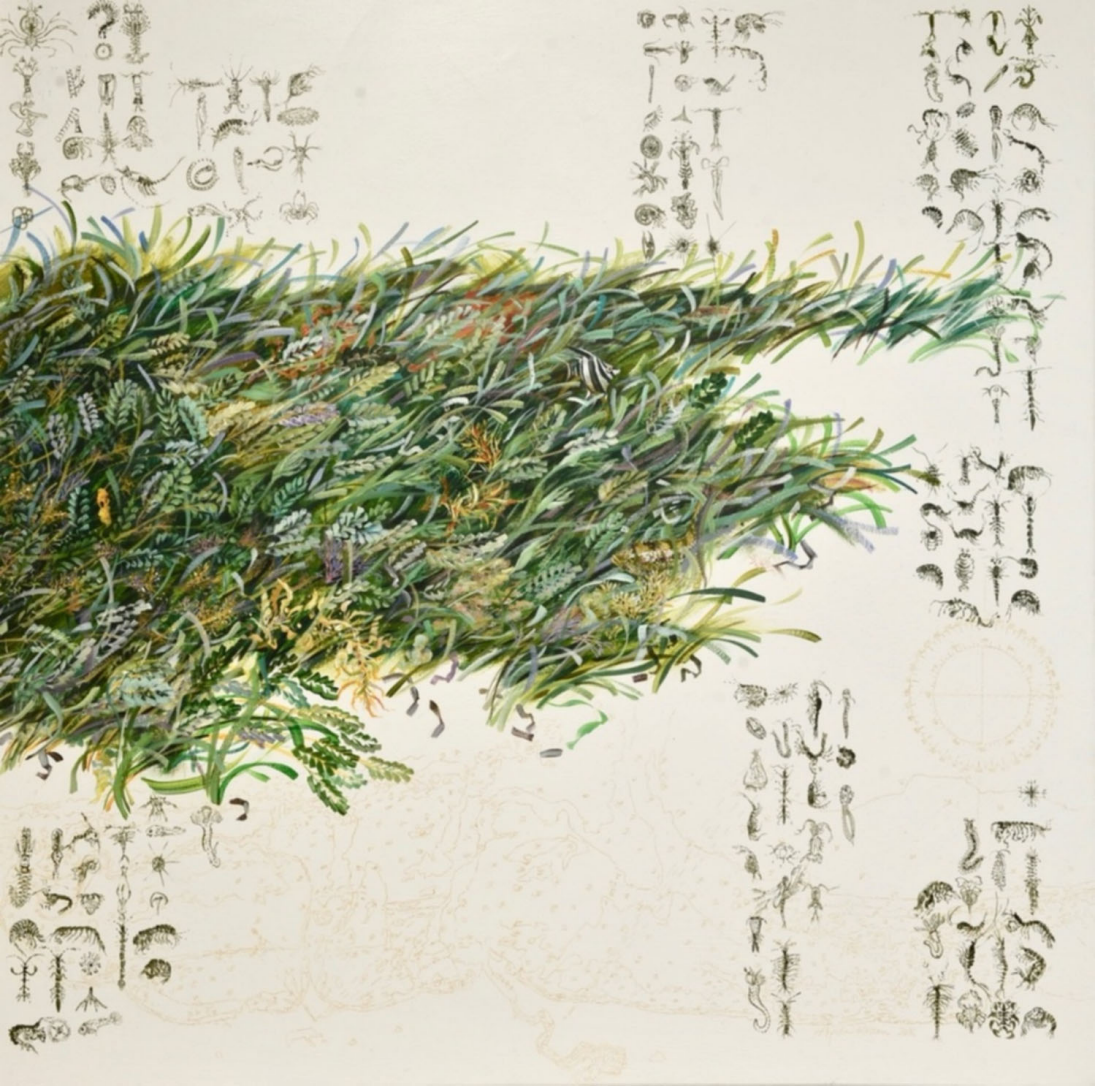
Artist, Angela Rossen’s artwork featuring seagrass. This piece is intended to communicate the issue of commercial exploitation of the waters above the seagrass meadows in Western Australia with no regard for the complex communities of plants and animals that live below. It features a benthic map of the seafloor in Western Australia and has a mariner’s compass on the right-hand side

Seagrasses have even been known to inspire art and be used as a medium to connect people with the marine environment (Fig. 8). The company ‘Rhythm in Bronze’ in Malaysia performed a special ensemble music piece in 2023 called “Seruan Setu: The Secret Gardens of the Sea” which was inspired by seagrass and the importance of protecting this habitat. Seagrasses have also featured in poems such as ‘The loves of plants’ by Erasmus Darwin and have their place in ancient legends and stories (Hines et al. 2005; Adulyanukosol et al. 2010) (see below), including mythologies, as is the case for the genus Posidonia which inherits its name from Poseidon, king of the sea.

### Ancient seagrass legend

*There is a legend in Thai culture that a pregnant woman who was suffering from morning sickness wanted to eat something different and so asked her husband to collect seagrass fruits for her. Being unsatisfied with the fruits collected for her, she ventured out to sea to pick the seagrass fruits herself. While standing out at sea, the tide came in and she could not walk to shore. She became trapped in the seagrass bed and turned into a dugong. Variations of the story say her husband followed his wife to live with her at sea. It is also said that the hands of the dugong and the fact they nurse their young is proof they used to be human* (Hines et al. 2005; Adulyanukosol et al. 2010).

### Seagrass poem

The following is an excerpt from the poem The Botanic Garden. Part II. The loves of the plants, by Erasmus Darwin, 1798:

*“Stretch'd on her mossy couch, in trackless deeps,*

*Queen of the coral groves, ZOSTERA sleeps;*

*The silvery seaweed matted round her bed,*

*And distant surges murmuring o'er her head.*

*High in the flood her azure dome ascends,*

*The crystal arch on crystal columns bends;*

*Roof'd with translucent shell the turrets blaze,*

*And far in ocean dart their colour'd rays;*

*O'er the white floor successive shadows move,*

*As rise and break the ruffled waves above.*

*Around the nymph her mermaid-trains repair*

*And weave with orient pearl her radiant hair;*

*With rapid fins she cleaves the watery way,*

*Shoots like a diver meteor up to day;*

*Sounds a loud conch, convokes a scaly band,*

*Her sea-born lovers, and ascends the strand.”*

Stories involving seagrass can also manifest in the passing of knowledge. The activities of invertebrate gleaning and harvesting of seagrass fruits is undertaken by women who bring their children to teach them how to collect these resources (Nessa et al. 2020). Stories are passed down to each new generation to educate on these practices and the value of seagrass beds (DSCP 2018). Gleaned invertebrates from seagrass beds are used in cultural activities such as weddings and funerals, and to play traditional games (Stiepani et al. 2023). Invertebrate gleaning is also known to be a social activity that enhances reconnection with nature and can be peaceful for the women involved (Grantham et al. 2020).

These harvesting activities are important for local communities, contributing to mental and physical well-being. Seagrasses provide this service to all communities at a broad scale, not just those who harvest from seagrass beds. Seagrasses naturally work to stabilise sediments and protect shorelines, enhancing the aesthetic appeal of coastlines. They also contribute to improving water quality and sanitation with growing evidence indicating that healthy seagrass meadows reduce the loads of pathogens, particularly those causing enteric diseases (Lamb et al. 2017, Tasdemir et al. 2024). Globally, this was calculated to avoid approximately 24 million gastroenteritis cases per year, which in turn was estimated to avoid treatment costs at around US$ 74 million per year (Ascioti et al. 2022). This shows the protective role seagrasses have beyond their direct use for medicinal purposes discussed above, which directly enhances human well-being. Seagrasses contribute to increasing the enjoyment people receive from coastal environments, encouraging people to be outdoors, reducing stress and overall supporting human happiness by helping people to reconnect with nature (Lloret et al. 2024).

## Valuing seagrass for their societal benefits

Humans and seagrass have had a rich history, and this interconnectedness is evidenced in the records of seagrass use in human societies. Reclaiming this history is an important step towards valuing seagrass for the societal benefits they provide. There are doubtless many more instances of seagrass featuring in human history that are buried in archives or in generational stories. Likely, a lot of historical information is hidden under the terms ‘seaweed’ or ‘algae’ instead of seagrass, not digitised, or in languages other than English, making it harder to locate these records of seagrass use. This is evidenced by the large geographic gaps identified in our review, namely Southern America and parts of Africa (Fig. 2a). Despite this, this study documents the major uses of seagrass throughout history, demonstrating the societal importance of this habitat.

Acknowledging human history is the first step to improving the societal valuation of seagrass and thereby, preventing overexploitation and major losses of seagrass habitat. We have an opportunity to learn from history. Humans spent thousands of years harnessing seagrass for different uses, from food to building materials, and while this worked well for some communities, on the global scale, human activities and ignorance of the importance of seagrass habitats have caused extensive seagrass loss. We can learn from past mistakes and bring attention to seagrass to further their appreciation, protection and sustainable use. Modern-day initiatives to re-integrate seagrass products into society helps to raise awareness and recognition for seagrass. While this is important for effective conservation and restoration today, it could be detrimental to seagrass longevity. Initiatives such as turning excess seagrass wrack into sustainable housing products instead of directing it to landfill, and establishing sustainable practice and regulation of seagrass harvest, are key to seagrass use in industry. It is important we raise awareness of seagrass for their societal values and benefits but ensure that this does not lead to overexploitation of seagrass habitat.

Honouring the historical and contemporary relationships between humans and seagrass helps to revalue seagrass from the bottom up, encouraging people to reconnect with this precious resource. By emphasising these relationships, cultural practices and well-being derived from seagrass, we can integrate seagrass societal values into ecosystem service frameworks. This will help to incentivise conservation efforts such that we do not lose these important habitats, and the customs associated with them. Human-seagrass relationships have persisted for thousands of years. It is important that we recognise this, and work to foster this relationship in modern society to promote better management plans that protect seagrasses for the future.

## Footnotes

1 www.tangtag.dk/
